# Safety and Efficacy of Transcatheter Arterial Chemoemboliazation in the Real-Life Management of Unresectable Hepatocellular Carcinoma

**DOI:** 10.5812/hepatmon.7070

**Published:** 2013-08-12

**Authors:** Argyro Mazioti, Nikolaos K. Gatselis, Christos Rountas, Kalliopi Zachou, Dimitrios K. Filippiadis, Kostantinos Tepetes, George K. Koukoulis, Ioannis Fezoulidis, George N. Dalekos

**Affiliations:** 1 Department of Radiology, Medical School, University Hospital of Larissa, University of Thessaly, Larissa, Greece; 2 Department of Medicine and Research Laboratory of Internal Medicine, Medical School, University of Thessaly, Larissa, Greece; 3 Department of Surgery, Medical School, University Hospital of Larissa, University of Thessaly, Larissa, Greece; 4 Department of Pathology, Medical School, University of Thessaly, Larissa, Greece

**Keywords:** Chemoembolization, Therapeutic, Carcinoma, Hepatocellular, Liver Cirrhosis, Hepatitis B Virus, Hepatitis C

## Abstract

**Background:**

Trans-arterial chemoembolization (TACE) is associated with better survival in BCLC-stage B patients with hepatocellular carcinoma (HCC) and Child-Pugh A whereas in Child-Pugh B there is no definite evidence of benefit.

**Objectives:**

To assess the safety and efficacy of TACE during routine clinical practice in a consecutive Greek cohort of patients with unrespectable HCC.

**Patients and Methods:**

Seventy one patients enrolled for this study (mean follow-up:24.6 months). 100 mg cisplatin, 50 mg doxorubicin and 10 ml lipiodol as well as embolic materials were used. CT-scans and blood tests were obtained prior and post-TACE. Kaplan–Meier method and Cox proportional hazard model were used to evaluate survival and factors affecting survival.

**Results:**

Survival at 1-year, 2-years, 3-years and 5-years was 73.2%, 45.4%, 33.2% and 14.9% respectively. Procedure-related mortality was 1.4%. Multivariate analysis showed lesion diameter, Child-Pugh classification, alcohol abuse, tumor response and AFP prior TACE as independent prognostic factors of survival. Patients diagnosed during surveillance had significantly better survival rates compared to those diagnosed after development of symptoms (HR = 0.58, 95%CI: 0.33-1.01, P < 0.05).

**Conclusions:**

TACE is safe and efficient for unrespectable HCC. Alcohol abuse, tumor burden, response criteria, Child-Pugh and AFP prior to the session were identified as independent predictors of survival whereas, adherence to surveillance programs resulted in significantly better survival in these patients.

## 1. Background

HCC is the third most common cause of tumor-related death among males and the sixth among females; without effective treatment the reported median survival is less than 5 months (1). In Greece, data from the HEPNET-GREECE Study Group has shown a cumulative HCC incidence approaching in 5 years 20% and 10% in decompensated and compensated HBV-related cirrhosis, respectively (2-4). In contrast, HCC incidence is less than 4% in HBV patients without cirrhosis whereas for HCV patients the incidence was even lower (1.4%) (2-4). Surgical resection and liver transplantation are the most effective treatments for early or very early HCC according to the BCLC staging-system (5, 6). However, many patients are presented with unrespectable HCC in the so-called intermediate stage according to BCLC (BCLC-stage B) where TACE is recommended as the standard care by many authorities (5-10). TACE seems to work well in prolonging the 2-year survival (OR in 35% of patients) particularly in BCLC-stage B patients with preserved liver function (Child-Pugh A) compared to the best supportive care or systemic chemotherapy (8, 11, 12). In HCC cases with Child-Pugh B, a case-by-case decision for TACE treatment seems mandatory, to be taken by multidisciplinary teams (8, 11, 12). In this context, several uncertainties have been raised in every day clinical practice regarding factors affecting TACE modality like tumor burden, selection criteria, chemotherapeutic regimens and use or disuse of lipiodol, use and the type of embolizing agents and the frequency of TACE courses. 

## 2. Objectives

Accordingly, the aim of the present study was to assess the safety and efficacy of TACE as well as the survival rates and the potential risk factors affecting survival during this routine clinicalpractice in a consecutive Greek cohort of patients with unrespectable HCC.

## 3. Patients and Methods

All participants included in this study were patients with unrespectable HCC who attended our clinic from 6/2003-9/2010 and consented to participate. Overall, 71 patients enrolled for TACE including 53/71 (74. 6%) with BCLC-stage B and 12/71 (16.9%) with BCLC-stage A in whom the risk of surgery or radiofrequency ablation was high. In addition, 2 had BCLC-stage C and 4 BCLC-stage D for whom TACE was provided because their performance status was 1-2; sorafenib was not available during that time, liver disease was stable without ascites and they had a solitary tumor. The clinical, epidemiological and demographic data of patients are shown in Table 1. HCC diagnosis was made according to EASL and AASLD ( 7 , 9 ). Accordingly, 37 patients had typical CT findings, 30 had two coincident imaging techniques and 4 had CT and positive biopsy under CT-guidance. Most patients (43/71; 60.6%) were asymptomatic and diagnosis was established during 6-monthly surveillances with AFP and ultrasound. In the remaining, HCC was diagnosed after admission for several reasons; e.g. highly elevated transaminases in a random check-up, abdominal pain, weight loss, etc.. Exclusion criteria included: extra hepatic metastases, active gastrointestinal bleeding, hepatic encephalopathy, refractory ascites or any known contraindication of TACE (i.e. impaired coagulation tests and renal failure). All subjects consented to participate by a written consent form. The ethical committee of the Medical School, University of Thessaly, Larissa, Greece approved the protocol. 

**Table 1.** Clinical, Epidemiological and Demographic Characteristics as Well as Response Rates According to RECIST and EASL Criteria in 71 Patients With Hepatocellular Carcinoma Treated by Trans-arterial Chemoembolization

	No. (%)
**Gender**	
Male	61 (85.9)
Female	10 (14.1)
**Age, y, Mean ± SD**	67.7 ± 9.4
**Autoimmune Hepatitis**	
No	69 (97.2)
Yes	2 (2.8)
**Alcohol abuse**	
No	41 (57.7)
Yes	30 (42.3)
**Chronic Hepatitis B**	
No	31 (43.7)
Yes	40 (56.3)
**Chronic Hepatitis C**	
No	60 (84.5)
Yes	11 (15.5)
**Primary Biliary Cirrhosis**	
No	69 (97.2)
Yes	2 (2.8)
**HCC^a^**	
Solitary	37 (52.1)
Multinodular	34 (47.9)
Lesion diameter, cm, Mean ± SD	7.4 ± 5.5
Number of sessions, Mean ± SD	2 ± 2
**Child-Pugh classification**	
Α	47 (66.2)
Β	20 (28.7)
C	4 (5.6)
**Response rates (RECIST^a^)**	
CR^a^	6 (8.5)
PD^a^	6 (8.5)
PR^a^	20 (28.2)
SD^a^	39 (54.9)
**Response rates (EASL^a^)**	
CR	23 (32.4)
PD	6 (8.5)
PR	12 (16.9)
SD	30 (42.3)
**Objective response (OR) according to RECIST**	
OR^a^	26 (36.6)
NR^a^	45 (63.4)
**Objective response (OR) according to EASL**	
OR	35 (49.3)
NR	36 (50.7)

^a^Abbreviations: CR, complete response; EASL, European association for the study of the liver; HCC, hepatocellular carcinoma; NR, non-responders; OR, Objective response; PD, progressive disease; PR, partial response; RECIST, response evaluation criteria in solid tumors; SD, stable disease

### 3.1. Imaging and Laboratory Studies

Dynamic CT-scan of the liver was performed for all patients prior and one month post TACE. For the evaluation of tumor response, tumor diameters as well as the percentage of viable (enhancing) tumor were taken into account according to the RECIST and EASL criteria (9, 13). An area that retained lipiodol for over one month was considered necrotic. All CT-scan evaluations were done by two radiologists (AM and CR). Standard laboratory markers including complete blood counts, coagulation tests, creatinine, urea and liver function tests (AST, ALT, γ-GT, LDH, ALP, total and direct bilirubin) were determined 1-day prior and 2-days post TACE. AFP was determined according to our 6-monthly surveillance program but also 1-day prior and one month post TACE.

### 3.2. TACE

A mixture of 100mg cisplatin, 50mg doxorubicin and 10ml lipiodol together with 10-15ml of non-ionic, water-soluble contrast material was used for all patients. In cases of multinodular disease in one hepatic lobe, a lobar chemoembolization was performed at the level of right/left hepatic artery, whereas for single lesions a selective or superselective approach was done. Embolic agents (embospheres) were used only in cases of selective/superselective chemoembolization (18 patients) in order to minimize the potential damage of the non-tumorous liver. TACE sessions were repeated until the tumor became completely necrotic. In fact, repetitions of TACE were performed in two-month intervals on the basis of tumor response on CT and patient tolerance that were assessed before any new course. Unsuccessful TACE treatment was considered when tumor growth or appearances of new tumors were observed after appropriate number of TACE sessions. The survival rate of patients was calculated from the date of first TACE (follow-up stopped 9/2010; mean follow-up 24.6 months). TACE-related death was considered as any death that occurred within 60 days of the session.

### 3.3. Statistical Analysis

Results are expressed as mean ± SD or SE and median-IQR, where appropriate. Data were analyzed by Student's t-test, Mann-Whitney U, paired t-test, Wilcoxon sign rank test, ANOVA, multivariate survival analysis using Cox proportional-hazard models with 95% CI and Kaplan-Meier survival analysis, where applicable. A two-sided p value of less than 0.05 was considered statistically significant.

## 4. Results

Most patients (43/71; 60.5%) were discharged 2-days after TACE. In total, 153 TACE procedures were performed (average number/patient: 2.16). In the majority of patients (34/71; 47.9%) one TACE session was done, in 18 (25.3%) two, in 9 (12.7%) 3, in 4 (5.6%) 4 and in the remaining 3 patients (4.2%) 5, 6 and 11 sessions were done in each of them respectively. TACE-related mortality was 1 out of 71 (1.4%). Post-embolization syndrome as attested by mild to moderate abdominal pain, low-grade fever and acute phase response (e.g. leukocytosis, elevation of transaminases and C-reactive protein) was observed in all patients, yet in all but one, the syndrome was lelf-limited. Severe liver decompensation and abdominal pain was observed in only one patient with multinodular HCC and partial portal vein thrombosis. This patient was hospitalized for 13 days and died 1.5 months after the procedure. Tumor response rates according to RECIST and EASL are shown in Table 1. Patients were classified as having PR, SD, CR, PD or NR (defined as SD plus PD; Table 1). The OR rate was defined as CR plus PR (Table 1) whereas, disease control rate was defined as OR plus SD (91.6% and 91.5% according to EASL and RECIST, respectively). The changes in laboratory parameters before and after TACE and according to the treatment response (OR vs. NR) are shown in (Appendixes 1, 2 and 3). Patients with OR demonstrated a significantly higher increase of bilirubin compared to that found in NR whereas, AFP decreased in OR but not in NR (Appendixes 1, 2 and 3).

### 4.1. Survival

**Figure 1.**
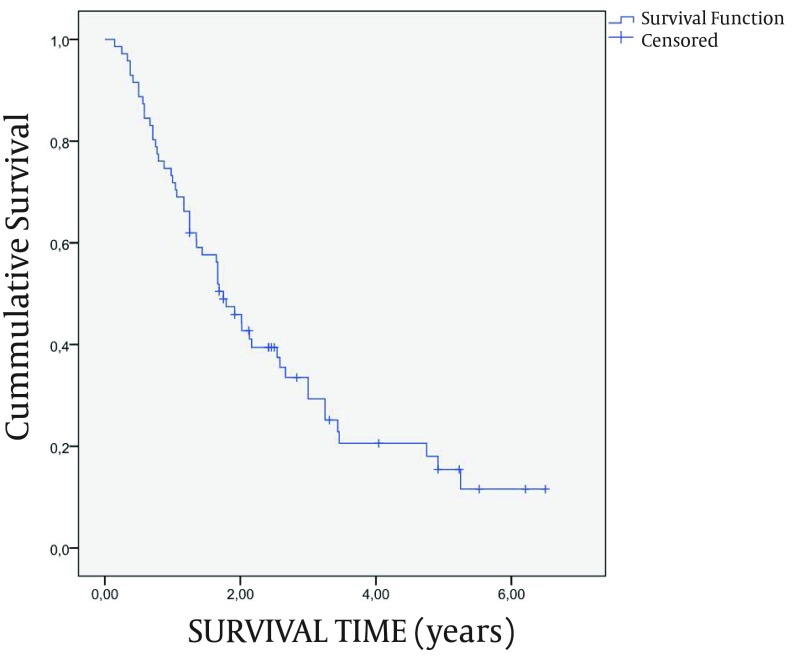
Survival rates of the patients according to Kaplan-Meier method. The cumulative survival rates at 6 months, 1-year, 2-years, 3-years and 5-years were 91.6% (SE = 3.3%), 73.2% (SE = 5.3%), 45.4% (SE = 6.0%), 33.2% (SE = 5.9%) and 14.9% (SE = 5.1%), respectively. SE = standard error

By the last follow-up 76.1% of patients had died. Mean survival was 2.5 years (SE = 0.3). The survival curve is shown in Figure 1. The use of embospheres did not affect the response rates and survival (data not shown). The main causes of death were: progressive liver failure (27 patients), variceal bleeding (5 patients), sepsis (4 patients) and non-liver-related reasons in the remaining patients. Univariate analysis revealed that multinodular HCC, lesion diameter, Child –Pugh classification, alcohol abuse, liver function tests, AFP prior TACE and RECIST and EASL classification were predictive prognostic factors of survival (data not shown). However, after multivariate survival analysis only the lesion diameter, Child-Pugh classification, alcohol-related cirrhosis, tumor response according to EASL criteria and AFP prior the session were independent prognostic factors of survival (Table 2). Survival was not affected by the BCLC B or BCLC A stage of patients (HR = 1.30, 95% CI: 0.61-2.78, P = 0.498; Supplement 1). On the contrary, hazard was significantly reduced for patients diagnosed during the screening surveillance compared to those diagnosed after the development of symptoms (HR = 0.58, 95% CI: 0.33-1.01, P < 0.05; Supplement 2).

**Table 2.** Multivariate Analysis of Independent Prognostic Factors of Survival

	HR^a^(95% CI )^a^	Ρ value
**Lesion diameter, cm**	1.15 (1.08-1.23)	< 0.001
**Child-Pugh classification**		< 0.001
A	1.00	
B/C	5.94 (2.58-13.66)	
**Alcohol abuse**		0.050
No	1.00	
Yes	1.88 (1-3.56)	
**EASL^a^**		0.022
OR^a^	1.00	
NR^a^	2.2 (1.12-4.33)	
**AFP^a^prior TACE^a^**	1.01 (1-1.01)	0.016

^a^ Abbreviations: AFP, alpha fetoprotein; EASL, European association for the study of the liver; HR, hazard ratio; NR, non-responders; OR, Objective response; TACE, Trans-arterial chemoembolization; 95%CI, confidence intervals

## 5. Discussion

The present study demonstrated that TACE was safe and well-tolerated in every-day clinical practice in a Greek cohort of patients with unresectable HCC. The mean cumulative 1-, 2-, 3- and 5-years survival rates were similar with two previous systematic reviews (8, 14). However, direct comparison of our results with previous reports (8, 14) cannot be done in a precise way since the results of these meta-analyses have been derived from studies that widely vary on the basis of patient selection criteria. Indeed, only selected patients are usually included in research trials, which is completely different from HCC patients encountered in routine clinical practice as studied in our report (12). Therefore, we tried to compare our results under real-life conditions with those revealed from studies using exactly or approximately the same procedures to our study. We found 6 studies in the English literature that completed the above mentioned criteria (15-20). In these studies including an overall 372 patients the reported 1-year, 2-years and 3-years survival rates were 50-75%, 23.3-59% and 14.8-41%, respectively which is in accordance with our findings. Regarding the hematological and biochemical markers, there was no significant change between prior and post TACE values when our patients were assessed according to the EASL criteria of response. This finding suggests that in patients with unresectable HCC who underwent TACE, the laboratory values prior and two days post the procedure cannot provide a preliminary hint concerning the likelihood of response to treatment. After multivariate analysis of alcohol abuse, lesion diameter, Child–Pugh classification, AFP level prior to the session and EASL classification were identified as independent prognostic factors of survival. Previous articles have already reported on the role of Child-Pugh classification, tumor size, AFP prior TACE and the proportion of tumor necrosis in the prediction of treatment response and survival (13, 21, 22). To the best of our knowledge, alcohol abuse has not been identified as an independent predictor of treatment response and survival in patients with unrespectable HCC. Therefore, further studies seem necessary in order to define if in fact alcohol abuse is a negative prognostic factor affecting treatment response and ultimately survival. Last but not least, we showed that the adherence to surveillance program for HCC diagnosis affected positively the survival of the patients. In conclusion, we showed that TACE is a safe and efficient technique for the management of patients with unrespectable HCC under real-life conditions. Alcohol abuse, tumor burden, response rate criteria, Child-Pugh classification and AFP prior to the session were identified as independent predictors of survival. Adherence to surveillance programs for HCC diagnosis seems to carry significantly better survival in patients diagnosed during the screening surveillance than those diagnosed after the development of symptoms.

## Supporting Information

Supplement 1. Kaplan-Meier Survival Estimates According to BCLC Stage

Supplement 2. Kaplan-Meier Survival Estimates for Patients Diagnosed During the Screening Surveillance Program and Patients Diagnosed After the Development of Symptoms

## Appendix

**Appendix 1.** Laboratory Findings Prior and Post TACE

	PRIOR TACE ^a^ (n = 71)		POST TACE (n = 71)		P value
	Mean ± SD	Median (median range)	Mean ± SD	Median (median range)	
**INR ^a^**	1.2 ± 0.3	1.2 (1-1.4)	1.3 ± 0.3	1.3 (1.1-1.5)	< 0.001
**Urea, mg/dL**	38.5 ± 13.7	36 (28-47)	41 ± 12	43 (32-51)	0.363
**Creatinine, mg/dL**	0.9 ± 0.2	0.9 (0.8-1)	0.9 ± 0.2	0.9 (0.8-1)	0.923
**Total protein ,g/dL**	7.5 ± 0.7	7.5 (7-7.9)	6.9 ± 0.7	6.9 (6.4-7.6)	< 0.001
**Albumin, g/dL**	3.7 ± 0.6	3.8 (3.3-4.2)	3.4 ± 0.5	3.4 (3.1-3.8)	< 0.001
**Total bilirubin, mg/dL**	1.6 ± 1.6	1.1 (0.7-2.1)	2.4 ± 2	1.7 (1.2-2.8)	< 0.001 ^b^
**Direct bilirubin, mg/dL**	0.4 ± 0.5	0.3 (0.2-0.5)	0.6 ± 0.6	0.4 (0.3-0.9)	< 0.001 ^b^
**AST ^a^, U/L**	59.5 ± 38.1	45.5 (32.5-84.5)	137 ± 105	105 (77-164)	< 0.001 ^b^
**ALT ^a^, U/L**	45.7 ± 33.3	33 (22-57)	96 ± 109	62 (44-98)	< 0.001 ^b^
**γ-GT ^a^, U/L**	120.3 ± 121.4	78.5 (35-158.5)	130 ± 165	82 (35-169)	0.013 ^b^
**ALP ^a^, U/L**	117.6 ± 49.7	114 (84-137)	115 ± 95	98 (75-122)	< 0.001 ^b^
**LDH a, U/L**	214.8 ± 120.9	192 (164-228)	279 ± 103	256 (207-321)	< 0.001 ^b^
**AFP ^a^, ng/mL**	1206.6 ± 3890.8	41.6 (4.5-195.9)	680.5 ± 2992.4	27.6 (4.5-94.0)	0.399 ^b^
**WBC ^a^, x 10^3^/ mm^3^**	6.2 ± 3.2	5.6 (4.3-7.3)	7.6 ± 3.2	7.1 (5.3-9.2)	< 0.001
**HCT ^a^, %**	38 ± 6.4	39 (34.4-42.6)	36.3 ± 5.2	37.2 (31-40)	< 0.001
**PLT ^a^, x 10^3^/ mm^3^**	156.6 ± 79.5	141 (95.5-185)	132 ± 65	120 (80-160)	< 0.001

^a^ Abbreviations: AFP, alpha-fetoprotein; ALT, alanine-aminotransferase; AST, aspartate-transaminase; γ-GT, gamma-glutamyl transferase; HCT, hematocrit; INR, international normaized ratio; LDH, lactate dehydrogenase; PLT, platelets; TACE, trans-arterial chemoembolization; WBC, white blood cells

^b^ Statistical tests performed are paired t-test and Mann-Whitney U test

**Appendix 2.** Laboratory Findings Prior and Post TACE Classified According to EASL Criteria

		PRIOR TACE		POST TACE		CHANGE	P value ^c^	P value ^f^
	OR ^a^ (n = 35) NR ^a^ (n = 36)	Mean ± SD	Median (median range)	Mean ± SD	Median (median range)	Mean ± SD		
**INR ** ^**a**^	NR	1.2 ± 0.3	1.2 (1-1.3)	1.3 ± 0.3	1.2 (1.1-1.3)	0.1 ± 0.2	0.062	0.091
	OR	1.2 ± 0.2	1.1 (1.1-1.4)	1.4 ± 0.3	1.3 (1.2-1.5)	0.2 ± 0.2	< 0.001	
	P value	0.878		0.348				
**Urea, mg/dL **	NR	35.2 ± 11.3	33 (27-41)	44.1 ± 10.7	47 (35-50)	8.9 ± 12.2	0.006	0.011
	OR	41.4 ± 15	41.5 (30-52)	38.5 ± 11.9	36 (29-51)	-2.9 ± 15.7	0.327	
	P value ^b^	0.066		0.076				
**Creatinine, mg/dL**	NR	0.9 ± 0.2	0.9 (0.8-1)	0.9 ± 0.2	0.9 (0.8-1)	0 ± 0.2	0.626	0.550
	OR	1 ± 0.2	1 (0.9-1)	0.9 ± 0.2	0.9 (0.8-1)	-0.1 ± 0.2	0.685	
	P value ^b^	0.062		0.529				
**Total protein, g/dL**	NR	7.4 ± 0.6	7.4 (7-7.8)	6.8 ± 0.8	6.9 (6.3-7.4)	-0.6 ± 0.7	0.001	0.921
	OR	7.6 ± 0.8	7.6 (7.1-7.9)	7 ± 0.7	6.9 (6.4-7.6)	-0.6 ± 0.7	< 0.001	
	P value ^b^	0.241		0.350				
**Albumin, g/dL**	NR	3.6 ± 0.6	3.7 (3.3-4)	3.4 ± 0.5	3.3 (3.1-3.9)	-0.2 ± 0.3	< 0.001	0.411
	OR	3.8 ± 0.6	3.9 (3.3-4.3)	3.4 ± 0.6	3.4 (3.1-3.8)	-0.4 ± 0.4	< 0.001	
	P value ^b^	0.331		0.813				
**Total bilirubin, mg/dL**	NR	1.8 ± 1.7	1.2 (0.9-2.5)	2.5 ± 2.5	1.9 (1.1-2.7)	0.7 ± 1	0.001 ^e^	0.044 ^f^
	OR	1.5 ± 1.5	1.1 (0.7-1.8)	2.3 ± 1.5	1.7 (1.2-2.9)	0.8 ± 1	< 0.001 ^e^	
	P value ^b^	0.196 ^d^		0.641				
**Direct bilirubin, mg/dL**	NR	0.5 ± 0.6	0.3 (0.2-0.6)	0.6 ± 0.4	0.5 (0.3-1)	0.1 ± 0.2	< 0.001 ^e^	0.181 ^ f^
	OR	0.4 ± 0.5	0.2 (0.1-0.4)	0.7 ± 0.7	0.4 (0.3-0.7)	0.3 ± 0.2	< 0.001 ^e^	
	P value ^b^	0.059 ^d^		0.797 ^d^				

^a^ Abbreviations: INR, international normalized ratio; NR, non-responders; OR, objective response

^b^ Group effect (Student t-test)

^c^ Time effect (Paired t-test)

^d^ Group effect (Mann-Whitney)

^e^ Time effect (Wilcoxon)

^f^Repeated measurement analysis of variance (ANOVA)-time x group effect.

**Appendix 3.** Laboratory Findings Prior And Post TACE Classified According to EASL Criteria (Continued)

	OR ^a^(n = 35) NR ^a^ (n = 36)	PRIOR TACE ^a^		POST TACE		CHANGE	P value ^c^	P ^f^
		Mean ± SD	Median (median range)	Mean ± SD	Median (median range)	Mean ± SD		
**AST ** **^a^, U/L**	NR	64.9 ± 42.3	55.5 (37-87)	127.3 ± 89.1	90 (77-118)	62.4 ± 82.2	< 0.001 ^e^	0.146 ^f^
	OR	54.7 ± 34	38.5 (32-76)	145 ± 119.1	108 (75.5-172)	90.3 ± 115.5	< 0.001 ^e^	
	P value ^b^	0.288		0.568 ^d^				
**ALT ** **^a^, U/L**	NR	42.1 ± 26.4	31.5 (25-54)	71.5 ± 59	57 (44-72)	29.4 ± 49.3	0.002 e	0.298 ^f^
	OR	48.8 ± 38.5	34 (19-58)	118.6 ± 136.9	87.5 (47-141)	69.8 ± 134.7	< 0.001 ^e^	
	P value ^b^	0.111 ^d^		0.783 ^d^				
**γ-GT ** **^a^, U/L**	NR	146.5 ± 145	91 (43-227)	156.3 ± 137.9	99 (56.5-224)	9.8 ± 27.8	0.107 ^e^	0.501 ^f^
	OR	97.2 ± 92	61 (32-137)	106.9 ± 185.1	44 (32.5-103)	9.7 ± 111.2	0.055 ^e^	
	P value ^b^	0.142 ^d^		0.043 ^d^				
**ALP ** **^a^, U/L**	NR	131.3 ± 52	124 (92-143)	114.8 ± 47	114 (83-124)	-16.5 ± 16.1	< 0.001 ^e^	0.128 ^f^
	OR	105.4 ± 44.9	99 (72-130)	115.4 ± 123	89.5 (59-122)	10 ± 100.3	0.011 ^e^	
	P value ^b^	0.039		0.155 ^d^				
**LDH ** **^a^, U/L**	NR	200.7 ± 38.5	197 (173-228)	285.7 ± 107.9	269 (196.5-322)	85 ± 103.3	0.003 ^e^	0.202 ^f^
	OR	226.7 ± 160.6	181.5 (160.5-216.5)	273.2 ± 101.6	243 (209-321)	46.5 ± 110.7	0.001 ^e^	
	P value ^b^	0.338 ^d^		0.695				
**AFP ** **^a^, ng/mL**	NR	2319.8 ± 5459.6	85.7 (4-457.4)	1368.4 ± 4792.8	28.9 (6.2-379)	-951.4 ± 2539.4	0.184 ^e^	0.341 ^f^
	OR	224.4 ± 765.1	38.1 (5.3–142.8)	284.4 ± 952.6	27.6 (4.2-65)	1461 ± 5222	0.042 ^e^	
	P value ^b^	0.246		0.840 ^d^				
**WBC ** **^a^, ** **x10^3^/ mm^3^**	NR	6.3 ± 3.2	5.7 (3.7-7.5)	8.1 ± 2.9	7.6 (6.3-9.4)	1.8 ± 2.4	0.001	0.091
	OR	6.2 ± 3.2	5.2 (4.4-7.3)	7 ± 3.4	6.7 (4.7-7.7)	0.8 ± 2.1	0.112	
	P value ^b^	0.868		0.258				
**HCT ^a^, %**	NR	37.1 ± 7.4	38.7 (33-42.4)	36.3 ± 4.9	38.1 (32-40)	-0.8 ± 2.9	0.017	0.307
	OR	38.9 ± 5.2	39 (36-42.8)	36.3 ± 5.6	36.3 (30.4-41)	-2.6 ± 2.8	< 0.001	
	P value ^b^	0.268		0.957				
**PLT ** **^a^, ** **x10^3^/ mm^3^**	NR	158 ± 74.7	147 (105-175)	136.1 ± 61.1	121.5 (89-158)	-21.9 ± 39.8	0.037	0.207
	OR	155.4 ± 84.9	121 (94-185)	127.7 ± 69	115.5 (73-162)	-27.7 ± 59	0.005	
	P value ^b^	0.898		0.650				

^a^ Abbreviations: AFP, alpha-fetoprotein; ALT, alanine-aminotransferase; AST, aspartate-transaminase; γ-GT, gamma-glutamyl transferase; HCT, hematocrit; LDH, lactate dehydrogenase; PLT, platelets; TACE, trans-arterial chemoembolization; WBC, white blood cells

^b^ Group effect (Student t-test)

^c^ Time effect (Paired t-test)

^d^ Group effect (Mann-Whitney)

^e^ Time effect (Wilcoxon)

^f^ Time effect (Repeated measurement analysis of variance (ANOVA)-time x group effect)
